# Case Report: A pharmacist-led precision therapy framework for managing invasive fungal infection in CSF1R-Related leukoencephalopathy post Allo-HSCT

**DOI:** 10.3389/fphar.2025.1656503

**Published:** 2025-11-27

**Authors:** Jin Lu, Mengqi Jia, Xinghua Luan, Zhongqiu Zhang, Jingying Wu, Xiaojun Huang, Li Yang, Xincai Zhao, Miaomiao Zhou, Yao Fu, Quanjun Yang, Jianping Zhang, Li Cao, Cheng Guo

**Affiliations:** 1 Department of Pharmacy, Shanghai Sixth People’s Hospital Affiliated to Shanghai Jiao Tong University School of Medicine, Shanghai, China; 2 Neurology and Genetics Clinical Pharmacy Team, Shanghai Sixth People’s Hospital Affiliated to Shanghai Jiao Tong University School of Medicine, Shanghai, China; 3 Department of Clinical Pharmacy, Shanghai General Hospital, Shanghai Jiao Tong University School of Medicine, Shanghai, China; 4 Department of Neurology, Shanghai Sixth People’s Hospital Affiliated to Shanghai Jiao Tong University School of Medicine, Shanghai, China; 5 Department of Genetics and Rare Diseases, Shanghai Sixth People’s Hospital Affiliated to Shanghai Jiao Tong University School of Medicine, Shanghai, China; 6 Shanghai Neurological Rare Disease Biobank and Precision Diagnostic Technical Service Platform, Shanghai, China

**Keywords:** multidrug management, CSF1R-related hereditary leukoencephalopathy, allogeneic hematopoietic stem cell transplantation, hereditary diffuse leukoencephalopathy with spheroids, invasive fungal disease

## Abstract

**Introduction:**

Hereditary diffuse leukoencephalopathy with spheroids (HDLS), caused by CSF1R mutations, is a rare autosomal dominant leukodystrophy characterized by rapid neurological decline. Hematopoietic stem cell transplantation (HSCT) is a promising treatment, but the risk of post-transplant complications such as invasive fungal disease (IFD) remains underexplored. Microglial dysfunction in CSF1R-related disorder (CRD) may further impair host immune defense.

**Methods:**

We describe a Chinese male with a non-hotspot CSF1R mutation (c.2443-1G>C) who underwent allogeneic HSCT. A multidisciplinary team (MDT), including clinical pharmacists, implemented an individualized pharmacological strategy for antifungal management, guided by immune status, infection risk, pharmacokinetics, and next-generation pathogen diagnostics.

**Results:**

Despite prophylaxis with voriconazole and levofloxacin, the patient developed febrile neutropenia and otitis media by day +16. Empirical meropenem therapy was ineffective, prompting escalation to teicoplanin and caspofungin. Pulmonary infection developed; targeted sequencing of bronchoalveolar lavage identified Aspergillus flavus. Antifungal therapy was intensified with voriconazole, resulting in clinical resolution by day +70. Treatment was maintained with good response.

**Discussion:**

This case demonstrates the complexity of managing IFD in CSF1R-related disorder patients after HSCT. The interplay between systemic immunosuppression and intrinsic microglial dysfunction may heighten infection susceptibility. Precision antifungal therapy guided by multidisciplinary team expertise and pharmacological monitoring may improve outcomes in this rare and high-risk population.

## Highlights

- This case presents the first reported CSF1R-related leukoencephalopathy with a non-hotspot c.2443-1G>C mutation complicated by invasive fungal infection after allo-HSCT. Microglial dysfunction in CSF1R-related disorder may amplify infection risk by impairing systemic immune surveillance post-transplant. A multidisciplinary team implemented a precision antifungal strategy integrating pharmacokinetics, immunologic status, and next-generation sequencing (tNGS) for pathogen identification.
- Breakthrough Aspergillus flavus infection was successfully managed with early escalation of voriconazole and caspofungin, supported by TDM and organ function monitoring.
- This case underscores the importance of individualized, adaptive pharmacological approaches in managing rare, immunocompromised neurological conditions post-HSCT.

## Introduction

Hereditary diffuse leukoencephalopathy with spheroids (HDLS) is a rare, autosomal dominant leukodystrophy characterized by severe disability and high mortality. Identified pathogenic mutations include those in the autosomal recessive AARS2 gene (Wade and Lynch, 2024) and the colony-stimulating factor-1 receptor (CSF1R) gene, the latter is relatively rare. CSF1R-positive HDLS is classified as a CSF1R-related disorder (CRD). CRD typically manifests in early adulthood with a broad spectrum of clinical symptoms, including rapidly progressive neuropsychiatric deterioration, movement and gait disorders, apraxia, epilepsy, and cortical dysfunction, ultimately leading to death. The estimated prevalence of CRD ranges from 30 to 75 cases per million (Dulski et al., 2023). Based on the reported, approximately only 300 cases of CSF1R-RD reported to date (Wade et al., 2024).

A genetic and phenotypic study of CRD patients in China (Wu et al., 2024) showed that the disease commonly presents as parkinsonism (46.0%) or cognitive impairment (38.6%), with a mean age of onset of 40.75 ± 8.58 years and a rapid disease course, with an average duration from onset to death of 2.8 ± 1.2 years. Most disease-associated CSF1R mutations (79%) are located in the tyrosine kinase domain (TKD, amino acids 582–910),with c.2381T>C/I794T identified as a hotspot mutation in Chinese CRD patients (accounting for 16.3% of cases). These mutations abrogate CSF1R kinase activity triggering a pathological cascade of microglial dysfunction (Rademakers et al., 2011), neuroinflammation, and neurodegeneration (Martins-Ferreira et al., 2025)—with microglial dysfunction emerging as the core pathological feature of CRDs. This dysfunction not only impairs central nervous system immune surveillance but may also disrupt systemic immune homeostasis via the blood-brain barrier distinguishes CRDs from other leukodystrophies (Wu et al., 2025).

Hematopoietic stem cell transplantation (HSCT) has been shown to slow CRD progression to some extent (Mochel et al., 2019):donor-derived hematopoietic stem cells can differentiate into functional microglia, replacing CSF1R-mutated, dysfunctional microglia, yet the clinical impact of CRD’s intrinsic immune vulnerability on post-transplant complications remains understudied. Invasive fungal disease (IFD) is a well-recognized HSCT complication, but the unique interplay between CSF1R mutation-driven microglial dysfunction and post-transplant iatrogenic immunosuppression—and how this synergy amplifies IFD risk—has not been clarified.

To date, there have been only 8 cases worldwide of CRDs treated with allo-HSCT, all from our clinical team (Wu et al., 2025), with only one of these cases developing invasive fungal disease (IFD) as a post-transplant complication. Building on this, we herein report the present case for the first time. The core value of this study lies in documenting the previously unreported unique clinical scenario of “CSF1R non-hotspot mutation + allo-HSCT + post-transplant IFD”. More critically, for the complex condition of this case, precisely tailored and adaptively adjusted pharmacotherapy served as the central pillar in controlling IFD and ensuring the patient’s post-transplant recovery. Beyond outlining the clinical course, we focus on elaborating the rationale behind the formulation and implementation of this individualized medication regimen, aiming to provide actionable pharmacotherapeutic references for clinicians caring for high-risk CRD patients post-HSCT.

## Case presentation

**FIGURE 1:**
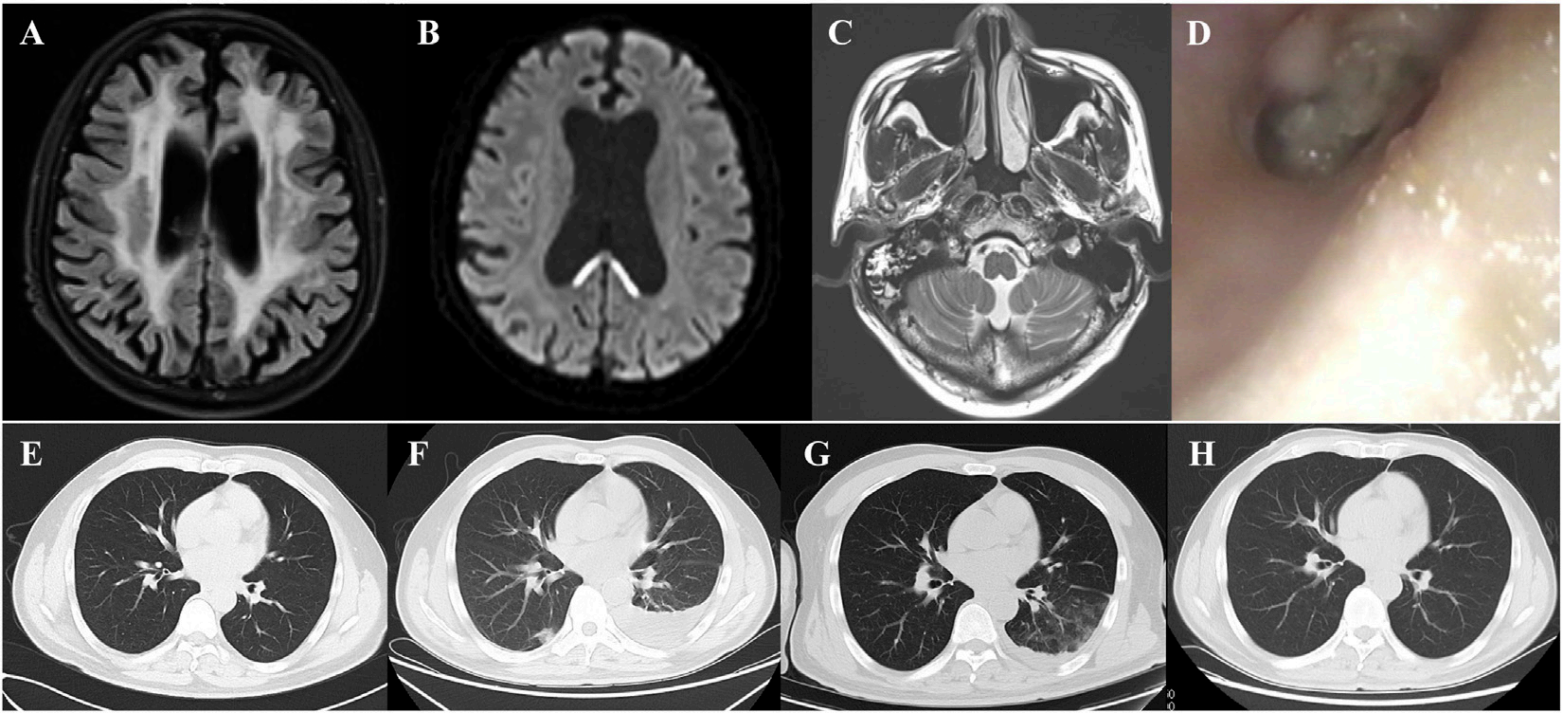
Radiographic and clinical findings during disease progression and infection events. **(A,B)** Brain MRI at initial presentation: T2-FLAIR images showing bilateral periventricular white matter hyperintensity and thinning of the corpus callosum, suggestive of hereditary leukoencephalopathy. **(C)** MRI of the temporal bone on day +16: inflammation of the right mastoid air cells consistent with otomastoiditis. **(D)** Otoscopic image showing purulent discharge in the right external auditory canal. **(E)** Chest CT on admission: no pulmonary abnormalities. **(F)** Chest CT on day +49: bilateral patchy infiltrates indicating fungal pneumonia. **(G)** Thoracentesis on day +63: turbid brown pleural fluid suggestive of fungal empyema. **(H)** Chest CT on day +85: resolution of prior lesions, with residual pleural effusion.

**FIGURE 2:**
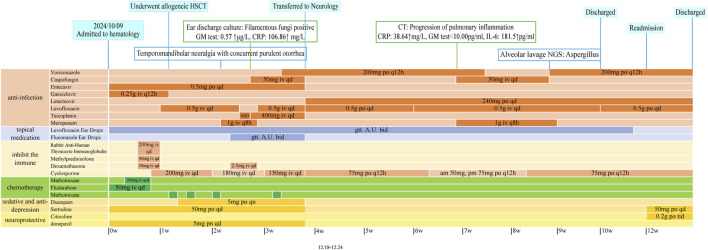
Timeline of clinical events, antimicrobial strategies, and pharmacological interventions. Detailed visualization of key timepoints from admission to discharge: including the day of hematopoietic stem cell transplantation (HSCT), onset of febrile neutropenia and ear infection, empirical and targeted antimicrobial treatments, pathogen identification via tNGS, adjustment of antifungal strategies, and recovery milestones. Drug regimens are color-coded to illustrate changes in anti-infective therapy and immunosuppressive management.

A 42-year-old male presented in May 2024 with sudden-onset dizziness, fatigue, and dysarthria. The brain MRI shows high signals around the periventricular white matter and in the tail of the corpus callosum, with thinning of the corpus callosum, suggesting a high likelihood of hereditary leukodystrophy (Figures 1a,b). Genetic testing confirmed a CSF1R mutation, diagnosing him with HDLS. On 9 October 2024, he was admitted to hematology and underwent allogeneic HSCT (9/10 HLA match) on post-admission day +10. Post-transplant prophylaxis included levofloxacin, voriconazole, and letermovir (due to a history of CMV infection), Details of all administered medications are provided in Figure 2. All concomitant medications used throughout the patient’s post-transplant period, including their dosages, administration durations, and key drug-drug interactions, are detailed in Table 1, which allows for a clear trace of the medication adjustment logic across different treatment phases.

**TABLE 1:** Concomitant medications, doses, durations, and key drug-drug interactions.

Treatment category	Drug	Dosage and administration	Date 09 Oct 2024–09 Jan 2025	Hospital day (+n)	Drug–Drug interaction
Anti-infection	Voriconazole	200 mg po q12h	Nov 03–29 Nov 2024; 10 Dec 2024–09 Jan 2025	+25 to +51; +62 to +93	Inhibits CYP3A4, CYP2C9, and CYP2C19; increases levels of cyclosporine, diazepam, and methylprednisolone
	Caspofungin	50 mg iv qd	Oct 28–03 Nov 2024; Nov 26–09 Dec 2024	+19 to +25; +48 to +61	Combined use with cyclosporine increases caspofungin exposure and may elevate liver enzymes
	Entecavir	0.5 mg po qd	Oct 09–03 Nov 2024	+0 to +25	
	Ganciclovir	0.25g iv q12h	Oct 09–16 Oct 2024	+0 to +7	
	Letermovir	240 mg po qd	01 Nov 2024–09 Jan 2025	+23 to +93	Decreases voriconazole levels via CYP2C9/2C19 induction; increases cyclosporine levels by CYP3A inhibition
	Levofloxacin	0.5g po qd	Nov 04–19 Nov 2024; 18 Dec 2024–09 Jan 2025	+26 to +41; +70 to +93	—
		0.5g iv qd	Oct 15–25 Oct 2024; Oct 29–03 Nov 2024; Nov 20–12 Dec 2024	+6 to +16; +20 to +25; +42 to +63	—
	Teicoplanin	600 mg iv qd 400 mg iv qd	27 Oct 2024; Oct 28–03 Nov 2024	+18; +19 to +25	—
	Meropenem	1g iv q8h	Oct 24–28 Oct 2024; Nov 26–10 Dec 2024	+15 to +19; +48 to +62	—
Topical medication	Levofloxacin ear drops	gtt. A.U. bid	Oct 08–19 Dec 2024	−1 to +71	—
	Fluconazole ear drops	gtt. A.U. bid	Oct 26–03 Nov 2024	+17 to +25	—
Inhibit the immune	Rabbit anti-human thymocyte immunoglobulin	200 mg iv qd	Oct 14–15 Oct 2024	+5 to +6	—
	Methylprednisolone	40 mg iv qd	Oct 14–15 Oct 2024	+5 to +6	Metabolized by CYP3A4; levels may rise with voriconazole or letermovir
	Dexamethasone	10 mg iv qd 2.5 mg iv qd	Oct 14–15 Oct 2024; Oct 26–28 Oct 2024	+5 to +6; +17 to +19	—
	Cyclosporine	200 mg iv qd×24h 180 mg iv qd×24h 150 mg iv qd×24h 75 mg po q12h am 50mg, pm 75 mg po q12h 75 mg po q12h	Oct 15–22 Oct 2024; Oct 23–29 Oct 2024; Oct 30–02 Nov 2024; Nov 03–22 Nov 2024; Nov 23–03 Dec 2024; 04 Dec 2024–09 Jan 2025	+6 to +13; +14 to +20; +21 to +24; +25 to +44; +45 to +55; +56 to +93	Levels increased by voriconazole and letermovir, therapeutic drug monitoring is essential. Additionally, cyclosporine is a P-gp inhibitor and may raise concentrations of P-gp substrates such as letermovir
	Human immunoglobulin injection (PH4)	10g iv qd 10g iv qd	Oct 16–18 Oct 2024; Jan 03–05 Jan 2025	+7 to +9; +87 to +89	—
Chemotherapy	Melphalan	300 mg ivgtt qd 200 mg ivgtt qd	12 Oct 2024; 14 Oct 2024	+3; +5	—
	Fludarabine	50 mg iv qd	Oct 09–13 Oct 2024	+0 to +4	—
	Methotrexate	0.027 iv st 0.018 iv st 0.018 iv st 0.018 iv st	18 Oct 2024; 20 Oct 2024; 23 Oct 2024; 31 Oct 2024	+9; +11; +14; +22	—
Sedative and anti-depression	Clonazepam	1 mg po q8h	Oct 12–14 Oct 2024	+3 to +5	
	Diazepam	5 mg po qn	Oct 19–04 Nov 2024	+10 to +26	Exposure increased by voriconazole; risk of oversedation, dose adjustment recommended
	Sertraline	50 mg po qd	Oct 09–04 Nov 2024; 30 Dec 2024–09 Jan 2025	+0 to +26; +82 to +93	—
Neuroprotective	Citicoline	0.2g po tid	30 Dec 2024–09 Jan 2025	+82 to +93	—
	Donepezil	5 mg po qd	Oct 09–04 Nov 2024	+0 to +26	—

On post-admission day +16, the patient developed fever and right ear pain with purulent discharge. Right otomastoiditis was diagnosed by MRI (Figure 1c) and otoendoscope (Figure 1d), and meropenem was initiated. On day +20, the culture of the discharge from the right ear grew filamentous fungi. Due to persistent fever, teicoplanin and caspofungin were added for broader coverage. By day +27, fever resolved, the patient transferred from hematology to neurology, and therapy was adjusted to levofloxacin, letermovir, and voriconazole.

On day +49, the patient developed a fever and the CT showed progression of the inflammation (Figure 1f), which compared with the chest CT on first admission day (Figure 1e), given his condition worsened and renal function, meropenem and cospofungin was restarted. On day +63, thoracentesis revealed turbid brown fluid (Figure 1g), and tNGS of BALF confirmed Aspergillus flavus. Emergency antifungal therapy with voriconazole was initiated, leading to rapid symptom improvement. By day +70, chest CT showed no new lesions, and pleural effusion decreased. On day +85, the patient was rehospitalized for an upper respiratory tract infection and treated with oseltamivir (Figure 1h). By day +96, he was afebrile and discharged.

## Conclusion

This case illustrates the challenges of managing invasive fungal disease in a patient with CSF1R-related leukoencephalopathy after allo-HSCT. The rare genetic mutation, combined with immunosuppression and CNS vulnerability, led to a highly complex medication profile. Our experience highlights the importance of precision multidrug management and close collaboration among clinical pharmacists and infectious disease specialists. Tailored antifungal therapy, guided by advanced diagnostics and pharmacokinetics, was key to clinical improvement. This case underscores the need for individualized treatment strategies in this rare, high-risk population.

## Discussion

This case report describes the medication-focused individualized application of existing precision tools for a patient with CSF1R gene mutation and leukodystrophy following allogeneic hematopoietic stem cell transplantation (allo-HSCT). Due to prolonged immune reconstitution and the use of immunosuppressive drugs post-transplant, invasive fungal disease (IFD) is one of the significant complications after HSCT. The incidence of IFD is relatively higher in allo-HSCT recipients (7.4%–13.1%) and has shown an increasing trend annually. The 1-year mortality rate for patients diagnosed with IFD post-HSCT ranges from 18.0% to 65.3% (Sun et al., 2015), highlighting the importance of early diagnosis and treatment, especially empirical drug decisions.

The main pathogens causing IFD are Aspergillus, followed by *Candida* and Mucor (Limper et al., 2011), among antifungal agents, voriconazole shows a higher response rate against invasive Aspergillus infections. Given that the fungal pathogen causing the patient’s ear infection remained unidentified initially, voriconazole was selected as the first-line broad-spectrum antifungal treatment.

Notably, during the treatment course, the patient’s IL-6 level surged to a peak of 181.5 pg/mL (normal range: 0–7 pg/mL)—a change that directly coincided with the progression of infection: the initial ear fungal infection extended to a secondary pulmonary fungal infection. To explain this association, a reasonable inference based on existing literature, including findings from our research team’s study published in Science (Wu et al., 2025) is that microglial dysfunction may be associated with the overexpression of pro-inflammatory genes (e.g., Fyn, Stat3)—accompanied by elevated levels of peripheral pro-inflammatory factors such as IL-6—and the underexpression of phagocytosis-related genes (e.g., Hck, Ptpn6). These abnormalities, in turn, can disrupt immune surveillance and trigger the spread of infections via the blood-brain barrier and systemic circulation.

It should be noted, however, that the current study did not perform microglial function assays or immunophenotyping on the patient; thus, further research is needed to validate this proposed mechanistic link. Despite this limitation, this case remains a rare report of a CRD patient with underlying microglial dysfunction who developed breakthrough IFD after allogeneic hematopoietic stem cell transplantation (allo-HSCT)—an outcome potentially tied to the aforementioned complex mechanisms.

Against this backdrop, and guided by the core pathological logic of “CSF1R mutations drive microglial dysfunction, which further heightens IFD susceptibility and may prolong post-transplant immune recovery,” we implemented individualized clinical management for the patient: we intensified IFD monitoring by increasing the frequency of galactomannan (GM) tests from the conventional once weekly to twice weekly, and extended antifungal maintenance therapy to post-transplant day +100 (a longer duration than the typical 6–8 weeks recommended for standard allo-HSCT recipients without CSF1R-related disorders).

As pulmonary infection progressed rapidly, high-risk patients must receive definitive diagnosis and aggressive antifungal treatment to reduce mortality (Busca et al., 2021), However, in this case, despite the use of conventional broad-spectrum antifungal therapy (typically covering Aspergillus), the GM test sensitivity decreased, and culture testing took a long time with a high false-negative rate. Microscopic examination was used for rapid presumptive diagnosis but could not accurately identify the fungal species, which is crucial for antifungal drug selection, as different species show different clinical manifestations. Targeted next-generation sequencing (tNGS) does not rely on physician assumptions and can effectively assist in diagnosing complex or mixed infections by testing pathogen genetic material in samples (Zhang et al., 2024). The patient’s culture and serological tests were negative, but tNGS of the bronchoalveolar lavage (BAL) fluid sample detected Aspergillus fumigatus with high sensitivity, indirectly confirming the diagnosis. Conversely, tNGS of the ear secretion sample did not detect any fungi, possibly due to the low pathogen load or interference from open cavities, leading to missed detection. This highlights the importance of considering infection site and sample type in tNGS testing, tNGS of BAL fluid provides more accurate pathogen identification for treating pulmonary infections.

The use of antifungal drugs with different mechanisms and monitoring of immunosuppressant levels played a key role in this case. For high-risk cases with monotherapy failure, multi-site infections, or drug-resistant fungi, the combination of antifungal agents with different mechanisms is recommended (Medical Mycology Society of Chinese MEducation, A., and Chinese Society of Hematology CMA, 2023), such as echinocandins combined with voriconazole, which may improve survival rates. In this case, following breakthrough *Aspergillus* infection, voriconazole and caspofungin were promptly used together. Given the patient’s declining renal function, meropenem dosage was adjusted from 0.5g q8h to 1g iv q12h.

**FIGURE 3:**
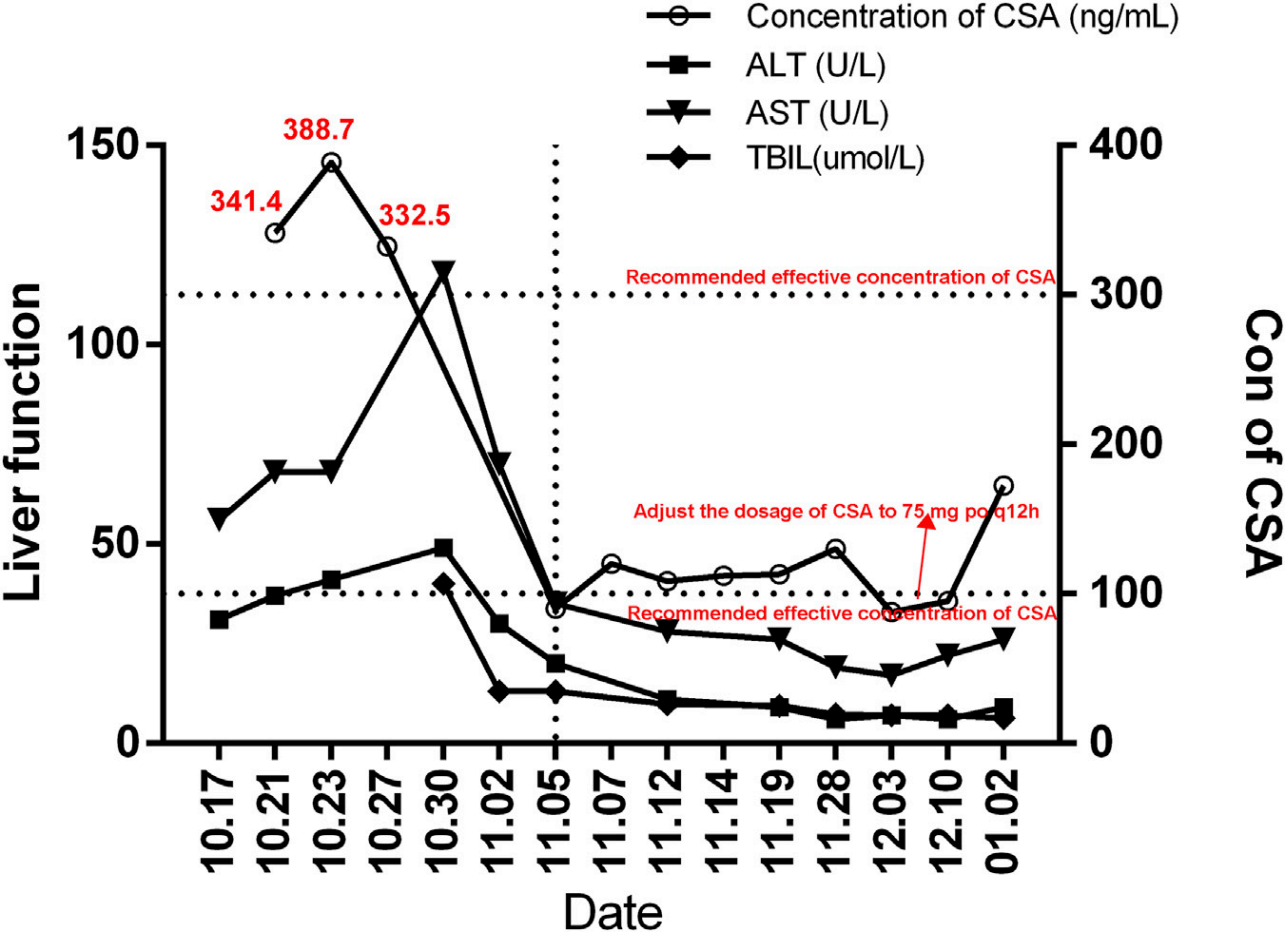
Monitoring Liver Function and Cyclosporine Levels to Guide Dosage Adjustment. The changes in the patient’s liver function indicators and cyclosporine blood concentrations were shown in Figure 2. These data were used to adjust the patient’s cyclosporine dosage to maintain therapeutic drug levels within the recommended range while monitoring liver function to assess potential hepatic effects of the medication.

**FIGURE 4:**
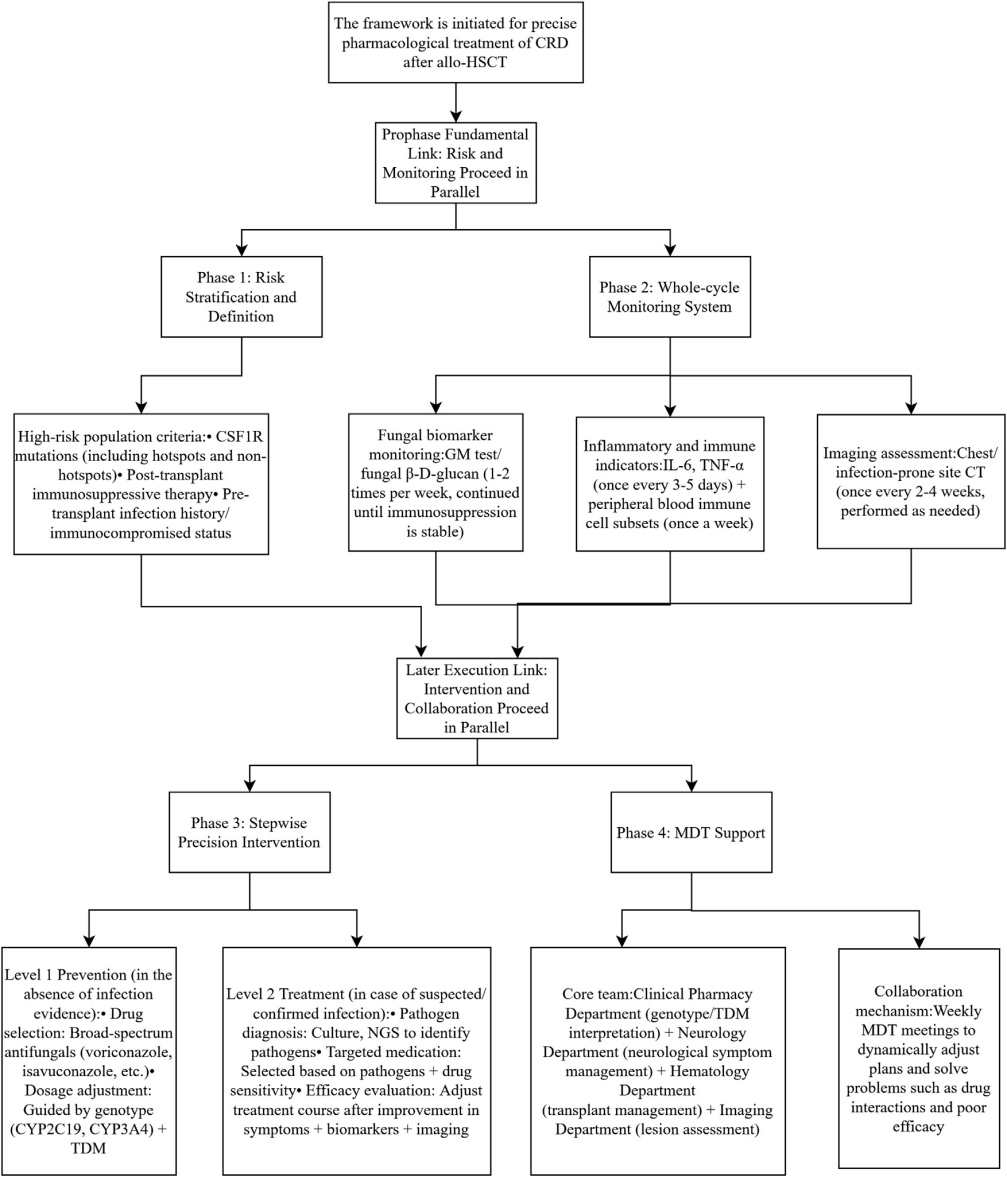
Pharmacist-Led Precision Intervention Framework. This figure illustrates the pharmacist-led precision intervention framework applied in the management of invasive fungal infection in CSF1R-related leukoencephalopathy after allo-HSCT. The framework integrates individualized pharmacological assessment, therapeutic drug monitoring, infection source control, drug–drug interaction evaluation, and multidisciplinary collaboration. Pharmacists play a central role in optimizing antifungal therapy through continuous evaluation of efficacy, safety, and pharmacokinetic parameters, ensuring a closed-loop precision therapy process that bridges clinical practice and translational pharmacology.

In this case, the combined use of therapeutic drug monitoring (TDM) and pharmacogenomic analysis enabled precise dose optimization of cyclosporine A and voriconazole. Post-transplant, the patient’s cyclosporine A trough levels consistently remained below the target range (87.75–130.35 ng/mL), which may be attributed to voriconazole’s inhibition of CYP3A4-mediated cyclosporine metabolism. Notably, although the patient carried the CYP2C19*1/*1 normal metabolizer genotype, the inhibitory effect of voriconazole on CYP3A4 still resulted in subtherapeutic cyclosporine exposure. Based on TDM data and dynamic liver function monitoring (Figure 3), we progressively adjusted the cyclosporine dose to 75 mg twice daily. This decision was made with reference to the recommended therapeutic window of 200–300 ng/mL in the post-HSCT setting. (Romero et al., 2002), Furthermore, this case highlights a critical therapeutic principle: when antifungal treatment inevitably alters the metabolism of immunosuppressants, maintaining effective drug concentrations through precise dose adjustments becomes essential. The precision pharmacotherapy management for invasive fungal infection in this post-transplant CRD patient was implemented based on the four-phase framework (Risk Stratification and Definition, Whole-cycle Monitoring System, Stepwise Precision Intervention, and MDT Support) illustrated in Figure 4, which provides a referable procedural paradigm for the diagnosis and treatment of similar complex cases. By detailing the clinical decision-making process, pharmacological strategies, and treatment outcomes, this report aims to offer practical guidance for the management of complex multidrug regimens and individualized pharmacotherapy in patients with CSF1R-related leukoencephalopathy following hematopoietic stem cell transplantation (HSCT).

## Long-term follow-up outcomes

As of the manuscript’s initial submission, the patient had completed 96 days of post-transplant follow-up and was discharged in a stable condition. To further evaluate the long-term efficacy of treatment and disease progression, we conducted an additional 3-month follow-up, extending the total post-transplant observation period to 186 days. The updated outcomes are summarized as follows: In terms of neurological function, the patient’s pre-transplant symptoms of dizziness and dysarthria were completely resolved; the Mini-Mental State Examination (MMSE) score, which was 24/30 at admission, improved to 28/30 at 186 days post-transplant, and no new movement or gait disorders emerged. For imaging findings, a brain MRI performed at 180 days post-transplant showed stable signal intensity in the periventricular white matter, with no progression of corpus callosum thinning; a chest CT scan confirmed the complete resolution of pleural effusion and fungal infiltrates that were previously present. Regarding antifungal therapy, maintenance treatment with voriconazole was discontinued at 120 days post-transplant due to the absence of invasive fungal disease (IFD) recurrence, and no long-term adverse events related to antifungal drugs (such as hepatotoxicity or visual disturbances) were observed during the entire follow-up period. These long-term follow-up data further confirm the effectiveness and safety of the individualized treatment strategy implemented for this patient, providing additional clinical evidence for the management of post-transplant complications in patients with CSF1R-related disorders.

## Data Availability

The original contributions presented in the study are included in the article/supplementary material, further inquiries can be directed to the corresponding authors.
